# High-intensity interval training differentially modulates acute BDNF and cognitive responses in young adult males: a randomized crossover trial

**DOI:** 10.1038/s41598-026-56739-4

**Published:** 2026-06-12

**Authors:** Yakup Zühtü Birinci, Serkan Pancar, Hasan Şimşek, Yusuf Soylu, Daniela Giconda Burac, Anca Ionescu, Şenay Şahin, Vlad Adrian Geantă

**Affiliations:** 1 Faculty of Sport Sciences, Bursa Uludağ University, Bursa, 16059 Turkey; 2 Faculty of Sport Sciences, Aksaray University, Aksaray, 68100 Turkey; 3 Faculty of Medicine, Aksaray University, Aksaray, 68100 Turkey; 4 Faculty of Sport Sciences, Tokat Gaziosmanpasa University, Tokat, 60250 Turkey; 5 Department of Physical Education and Sport, Stefan cel Mare University, Suceava, 720229 Romania; 6 Department of Motric Performance, Transylvania University of Brasov, Brașov, 600115 Romania; 7 Department of Physical Education and Sport, Faculty of Physical Education and Sport, Aurel Vlaicu University of Arad, Arad, 310330 Romania

**Keywords:** Brain-derived neurotrophic factor, Executive function, Exercise intensity, High-intensity interval training, Lactate, Brain health, Neurology, Neuroscience

## Abstract

Exercise is widely recognized for its beneficial effects on brain health, yet the extent to which exercise intensity modulates acute neurochemical and cognitive responses remains unclear. Particularly, the role of exercise intensity in shaping brain-derived neurotrophic factor (BDNF), lactate responses, and executive function requires further investigation. This study compared the acute effects of low-intensity continuous training (LICT), moderate-intensity continuous training (MICT), high-intensity interval training (HIIT), and a resting control condition (CTRL) on BDNF levels, blood lactate concentration, and cognitive responses in healthy young adult males. Twelve healthy young adult males completed LICT, MICT, HIIT, and the control condition using a randomized crossover design with a 7-day washout period. Serum BDNF, blood lactate concentration, and executive function assessed by the Stroop Test were measured before and immediately after each experimental condition. HIIT induced significantly greater post-exercise increases in BDNF and lactate compared with all other conditions, while MICT elicited moderate elevations relative to LICT and rest. Lactate responses increased progressively with exercise intensity. Improvements in executive function were observed exclusively following HIIT, reflected by significantly faster Stroop Test completion times. HIIT produced concurrent elevations in lactate and serum BDNF together with improved executive function performance. HIIT may represent an effective acute stimulus for cognitive benefits, with potential relevance for exercise approaches aimed at supporting brain health via neurotrophic signaling.

**Trial registration:** The study was retrospectively registered on ClinicalTrials.gov (identifier: NCT07137611; https://clinicaltrials.gov/study/NCT07137611) on 22 August 2025.

## Introduction

Exercise is increasingly viewed as an effective, non-pharmacological, affordable, and accessible strategy to enhance, maintain, and restore various domains of cognitive function across the lifespan^1,2^. Although a growing body of evidence has provided increasingly nuanced insights into how exercise benefits brain function, the mechanisms underlying exercise–brain crosstalk remain largely elusive^3,4^.

From an evolutionary standpoint, it has been hypothesized that the physical demands of endurance-based locomotion in ancestral environments, particularly those linked to subsistence activities such as persistence hunting and resource acquisition—acted as a selective pressure promoting neurocognitive adaptations through metabolic signaling cascades^5,6^. Converging evidence supports this view, showing that a single bout of exercise can elevate circulating neurotrophic factors that are putatively linked to enhanced synaptic plasticity, learning, and memory^7,8^.

Brain-derived neurotrophic factor (BDNF) is one of the most empirically investigated neuroprotective molecules, primarily due to its essential role in a broad range of neurophysiological processes^9^. Specifically, BDNF supports neurogenesis, synaptogenesis, and long-term potentiation, especially in brain regions involved in complex cognitive functions such as the hippocampus and prefrontal cortex^6^. Notably, cumulative evidence suggests that exercise is among the most potent physiological stimuli for enhancing both peripheral and central BDNF expression, thereby facilitating synaptic plasticity and improving cognitive performance^10,11^.

Another molecule, lactate, once considered a waste product of anaerobic metabolism, has emerged as a promising neuromodulator biomarker attracting increasing scientific attention^12^. Recent animal and human studies suggest that lactate acts as a signaling agent that might link exercise to BDNF-related neurobiological mechanisms^12–14^. Although several pathways have been proposed through which lactate may influence BDNF expression during and following exercise, these mechanisms are primarily supported by preclinical and indirect human evidence. Proposed pathways include activation of the sirtuin 1 (SIRT1)–peroxisome proliferator-activated receptor gamma coactivator 1-alpha (PGC1α)–fibronectin type III domain-containing protein 5 (FNDC5) cascade^13^. Additional mechanisms include modulation of intracellular calcium signaling via nicotinamide adenine dinucleotide (NADH) accumulation and binding to hydroxycarboxylic acid receptor 1 (HCAR1)^15,16^. Nevertheless, limited knowledge exists about which exercise variables (e.g., intensity, work interval duration, volume, session duration, work-to-rest ratio) optimize BDNF and lactate responses in healthy adults.

Exercise intensity represents a readily modifiable component of training and may be considered a key factor in the exercise–cognition literature, particularly as a moderator of neurobiological and behavioral outcomes^17–19^, though some reports indicate that the current evidence remains insufficient to draw definitive conclusions^2,20^. Numerous studies demonstrated that acute high-intensity interval training (HIIT) sessions in healthy adults have been associated with enhanced cognitive performance^21–23^, and elevated BDNF levels^24–27^. Moreover, recent syntheses of the literature has implicated elevated lactate levels, as a plausible mechanistic driver of these effects. Evidence suggests that lactate can cross the blood–brain barrier^28^ and stimulate BDNF release, promoting both neuroplasticity and cognitive performance improvements^3,29–32^.

Although these findings are promising, direct comparisons between HIIT and moderate‑ and low‑intensity exercise have not yet demonstrated a clear superiority of one modality over the other in enhancing cognition or neurochemical markers. Furthermore, a paucity of data precludes identification of the optimal exercise protocol for maximizing neurotrophic‑driven cognitive outcomes. In this context, given that lactate production scales with exercise intensity, defining exercise protocols that effectively target the lactate–BDNF crosstalk holds critical implications for precision‑based cognitive interventions. Therefore, this study compared the acute effects of different exercise intensities on serum BDNF, lactate responses, and cognitive performance in healthy young adult males. It was hypothesized that a single session of HIIT would produce significantly greater increases in serum BDNF and lactate levels, along with improvements in executive function performance, particularly on the Stroop interference task, compared with lower-intensity and control (CTRL) conditions.

## Materials and methods

### Research design

This study employed a randomized crossover controlled trial design to examine the acute effects of three different intensities of running exercise — LICT, MICT, and HIIT — as well as a resting CTRL session, on cognitive performance (assessed using the Stroop test) and neurochemical responses (e.g., serum BDNF and blood lactate levels) in healthy young adult males (18–25 years).

**Fig. 1:**
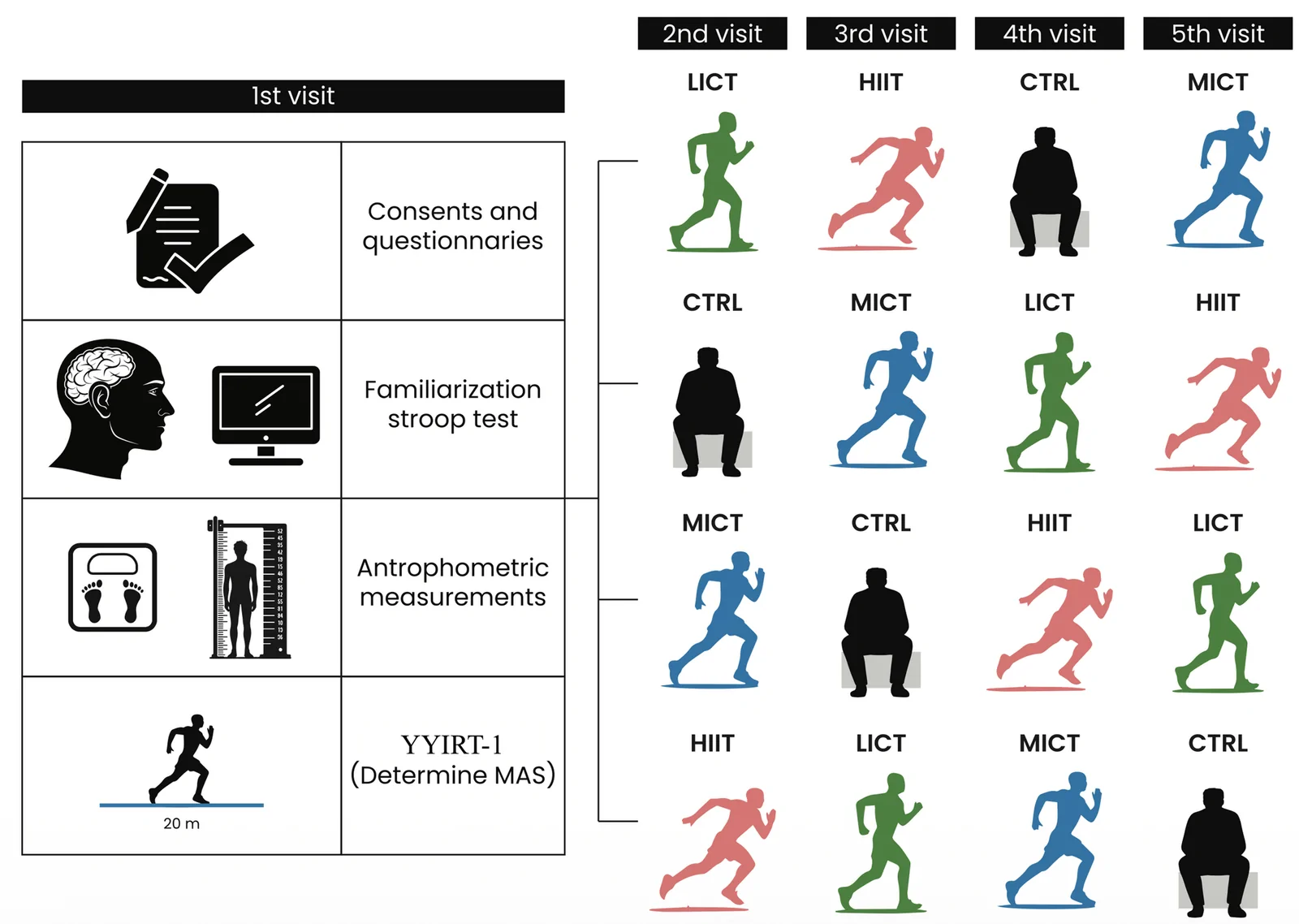
Study design. *CTRL* control, *HIIT* high-intensity interval training, *MICT* moderate-intensity continuous training, *LICT* low-intensity continuous training, *MAS* Maximum aerobic speed, *YYIRT-1* Yo-Yo Intermittent Recovery Level 1 Test.

Participants were assigned to one of four condition sequences using a computer-generated random sequence (https://www.randomizer.org) in a randomized, counterbalanced order with a 7-day washout between sessions to minimize residual physiological or cognitive effects. A Latin square design was used to ensure that each condition occurred equally across sessions (1–4), with systematic rotation of condition sequences to control for order effects. In addition to the washout period, HIIT and MICT were not scheduled in consecutive sessions (i.e., HIIT was never followed by MICT at the subsequent visit, and MICT was never followed by HIIT) to further reduce the likelihood of residual physiological or neurochemical effects (e.g., transient post-HIIT increases in BDNF reported by Skriver et al.^33^, (Fig. 1). Because HIIT and MICT were expected to impose greater physiological demands, they were separated by lower-load conditions (LICT or CTRL) whenever applicable, thereby reducing the likelihood of first-order carryover effects. At study completion, all participants had completed each condition once, ensuring both full counterbalancing and intensity balance across the sample.

### Participants

Sample size estimation was conducted using G*Power software (version 3.1.9.7; University of Düsseldorf, Düsseldorf, Germany) based on a repeated-measures ANOVA (within factors). The calculation was performed using the expected changes in serum BDNF, which was defined as the primary biological outcome of the study. The effect size was derived from the BDNF group × time interaction reported by Hung et al.^34^, with a partial eta squared of 0.33. This corresponds to an effect size (f) of 0.70. The analysis was performed with an alpha level of 0.05 and a statistical power of 0.80 (1-β = 0.80), assuming 4 conditions and 2 measurement points. The output parameters indicated a critical F value of 7.71 and a total required sample size of 8. However, the study was conducted with 12 participants.

Twelve healthy young adult males [age: 19.67 ± 0.9 years, height: 183.3 ± 5.1 cm, weight: 76.44 ± 8.3 kg; body mass index: 22.64 ± 1.6 kg·m⁻²; body fat percentage: 10.3 ± 1.3%; maximal heart rate (HRmax): 204 ± 21.2 bpm; MAS: 15.7 ± 1 km·h⁻¹; VO_2_max: 55.83 ± 3.31 mL·kg⁻¹·min⁻¹] voluntarily participated in this study.

Participants were recruited through announcements shared on social media platforms and posters displayed in public areas such as university campuses and sports centers. Individuals who expressed interest were invited to the laboratory for a detailed eligibility screening. Inclusion criteria required participants to be aged 18 years or older, be native Turkish speakers, have normal or corrected-to-normal visual acuity and normal color perception, possess valid medical clearance, have no history of significant lower limb injuries within the past year, and have not recently used any substances or supplements known to enhance aerobic or anaerobic capacity. Exclusion criteria included tobacco use, consumption of alcohol or recreational drugs, use of psychoactive medications, and a history of depression, neurological disorders, or neuromuscular or musculoskeletal conditions. Comprehensive verbal information about the study’s procedures, potential benefits, and risks was provided during the initial visit and reiterated before each familiarization and intervention session. Written informed consent was obtained from all participants prior to participation, and verbal confirmation of willingness to continue was obtained before each session.

**Fig. 2:**
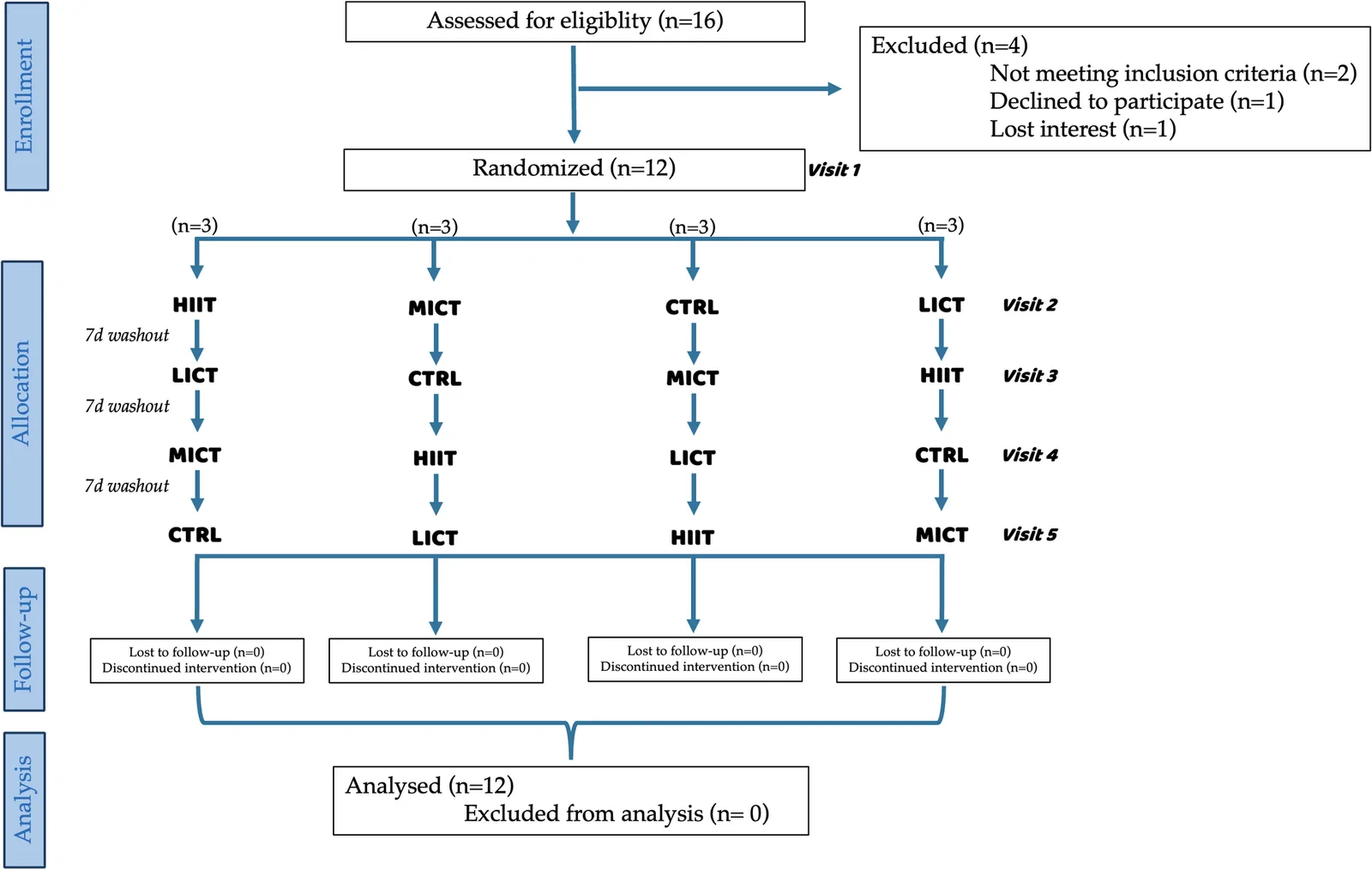
Consolidated Standards of Reporting Trials (CONSORT) flowchart of a four-arm randomized controlled cross-over and the number of participants lost to follow‐up. *CTRL* control, *HIIT* high-intensity interval training, *MICT* moderate-intensity continuous training, *LICT* low-intensity continuous training.

The overall study design and reporting were adhered to the Consolidated Standards of Reporting Trials (CONSORT)^35^ and the Standard Protocol Items: Recommendations for Interventional Trials (SPIRIT)^36^. Additionally, relevant CONSORT extensions for within-subject designs^37^ and non-pharmacological interventions^38^ were specifically considered (Fig. 2). Due to the nature of the study design, non-pharmacological within-subject design, blinding was not feasible. To minimize potential bias, participants were kept unaware of the study hypotheses, and blood samples were labeled with anonymized codes such that laboratory assays were performed by personnel blinded to condition. The study was approved by the Research Ethics Committee of Tokat Gaziosmanpaşa University (protocol code: TOGÜ.FRM.185-8/10, date of approval: 29 May 2025) and conducted in accordance with the Declaration of Helsinki. All participants provided written informed consent prior to participation.

### Procedures

All training sessions were supervised by a credentialed strength and conditioning professional to promote standardized delivery and protocol adherence and were conducted between 16:00 and 18:00 at a track-and-field facility to minimize circadian-related variability. A portable digital thermo-hygrometer was used prior to each session to document ambient temperature (22–25 °C) and relative humidity (35–45%), which remained within a consistent range across testing days. To standardize pre-session conditions, participants avoided strenuous exercise in the 48 h leading up to testing, did not consume alcohol within 24 h of each session, and slept for 7–8 h beforehand. Participants were instructed to maintain their habitual dietary patterns and general lifestyle throughout the study and to refrain from food and caffeine intake for at least 2 h before each testing session; however, macronutrient intake was not formally standardized.

At the initial visit, participants**’** body composition parameters were assessed, after which resting heart rate (HRrest) was recorded. To minimize potential learning effects, all participants then completed a Stroop Test familiarization session, followed by the Yo-Yo Intermittent Recovery Test Level 1 (YYIRT-1) to determine maximal aerobic speed (MAS), aerobic capacity, and maximal heart rate (HRmax).

**Fig. 3:**
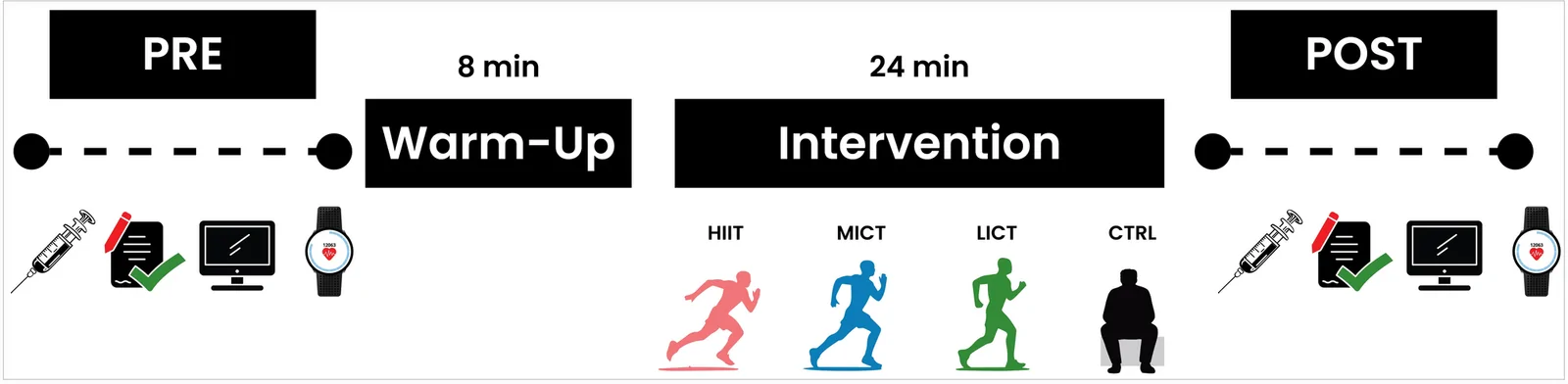
Experimental protocol design. *CTRL* control, *HIIT* high-intensity interval training, *MICT* moderate-intensity continuous training, *LICT* low-intensity.

Across the subsequent four visits, each participant performed every experimental condition. Each session commenced with a standardized 8-min warm-up comprising light jogging, dynamic stretching, and movement-preparation drills. All sessions were delivered on a standard track-and-field facility and lasted ~ 32 min in total, including warm-up and 24 min of exercise (Fig. 3).This exercise total duration was selected based on evidence that similar-duration exercise sessions tend to optimize executive function outcomes^18,39^. Running distances were calculated individually as speed × time using each participant’s MAS to standardize exercise prescription; sessions were matched by total duration across conditions, whereas total distance and physiological load were not intended to be equivalent.

Pre- and immediate post-session (≤ 1 min) venous blood samples were obtained to quantify neurochemical markers. Executive function was then assessed via the Stroop test to evaluate cognitive performance. Heart rate was recorded continuously throughout all exercise conditions, and perceived exertion was rated using Borg’s 6–20 rating of perceived exertion (RPE) scale^40^.

#### Exercise ınterventions

**Fig. 4:**
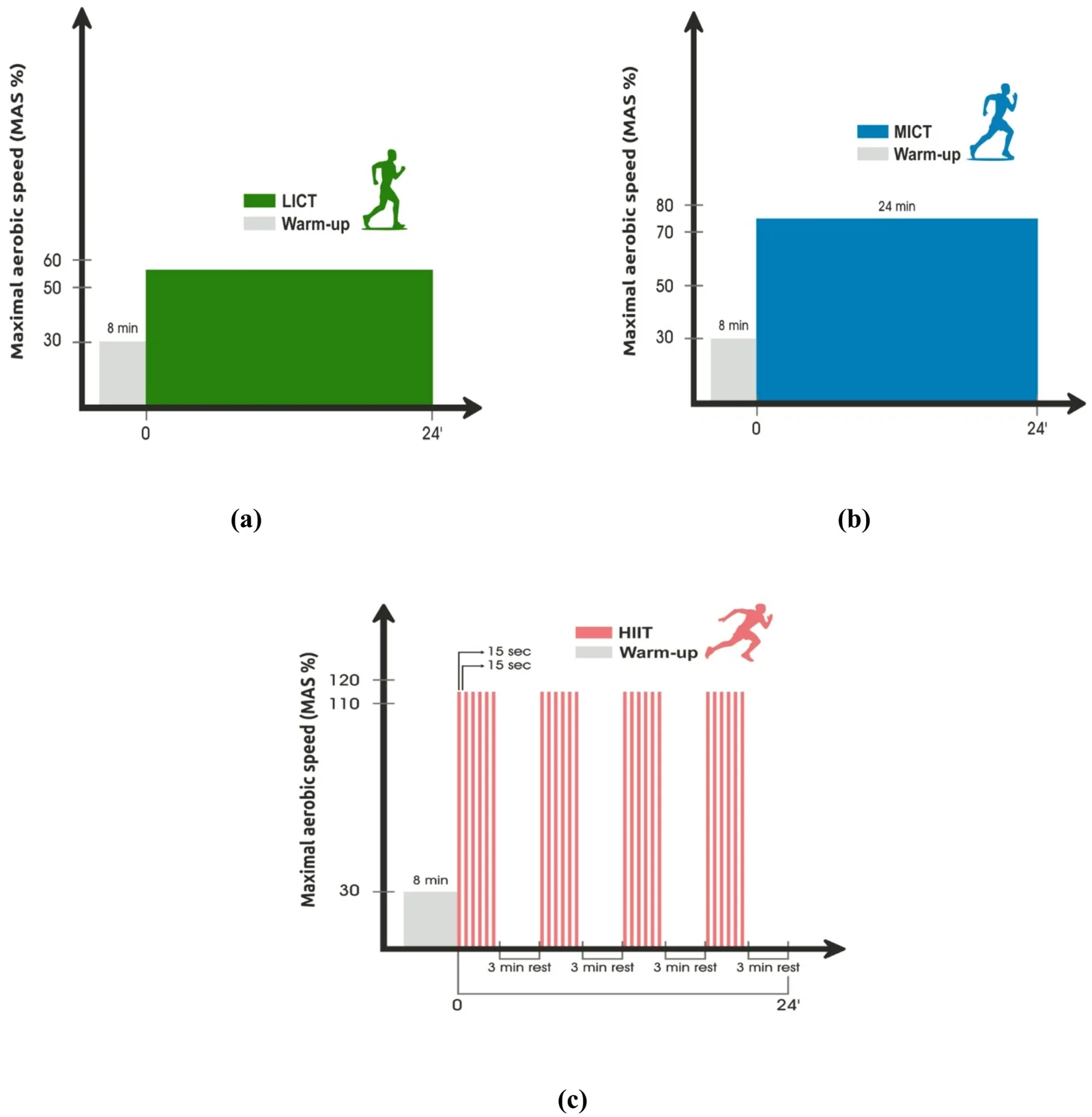
Exercise interventions. (**a**) Low-intensity continuous training (LICT) (**b**) Moderate-intensity continuous training (MICT). (**c**) High-intensity interval training (HIIT). sec: second; min: minute.

In the Table 1; Fig. 4 characteristics of the experimental sessions were given.

**Table 1:** Characteristics of the exercise and control sessions.

Session	No. x duration of bout	Work velocity	No. x duration of series	Intraseries recovery	Interseries recovery	Total duration
HIIT	6 × 15 s	110–120% MAS	4 × 3 min	15 s passive	3 min passive	24 min
MICT	1 × 24 min	70–80% MAS	1 × 24 min	**−**	**−**	24 min
LICT	1 × 24 min	50–60% MAS	1 × 24 min	**−**	**−**	24 min
CTRL	No exercise/resting					24 min

*CTRL* control, *HIIT* high-intensity interval training, *MAS* maximal aerobic speed, *MICT* moderate-intensity continuous training, *LICT* low-intensity continuous training.

LICT: Participants ran continuously for 24 minutes at 50–60% of their MAS without rest (Fig. 4a).

MICT: Participants completed a continuous 24-min running bout at 70–80% of their MAS. The exercise was performed at a steady, uninterrupted pace throughout the session, with no planned rest periods. Participants were instructed to maintain the prescribed pace for the entire duration (Fig. 4b).

HIIT: Participants performed 15-s running bouts at 110–120% of MAS, with the target distance for each bout pre-calculated from individual MAS (speed × time). Running was conducted shuttle-style between two cones. At the whistle, participants ran from one cone to the other for 15 s and then remained standing still for a 15-s passive rest at that cone. At the next whistle, they ran back in the opposite direction, followed by another 15-s stationary recovery. This 15-s run/15-s recovery cycle continued for 3 min, constituting one set (6 runs and 6 recovery intervals per set). Four sets were completed, separated by 3-min passive inter-set rest, resulting in a total session duration of 24 min. In total, the HIIT protocol included 6 minutes of active running and 18 minutes of passive recovery (Fig. 4c).

CTRL: Participants remained seated at rest for the same duration (24 minutes), under the same environmental conditions and time schedule as the exercise conditions.

### Measurements

#### Anthropometric assessments

All anthropometric assessments were conducted in the morning before breakfast. Participants’ stature was measured and body mass were assessed using a body composition analyzer (BC-418MA, Tanita Corp., Tokyo, Japan). The instrument uses multi-frequency bioelectrical impedance analysis, enabling a detailed assessment of body-composition–related parameters.

#### Yo-Yo Intermittent Recovery Test level 1 (YYIRT-1)

To determine aerobic performance, participants completed the YYIRT-1, a validated, progressively intensifying test guided by audio signals, in accordance with the protocol developed by Bangsbo and colleagues^41^. The highest HR value recorded during the test was defined as the participant’s HRmax.

#### Estimation of maximum oxygen uptake (VO_2_max)

Because direct cardiopulmonary measurement was not performed, VO₂max was estimated from YYIRT-1 performance according to the prediction equation described by Bangsbo et al.^41^

VO₂max (mL·kg^−1^·min^−1^) = YYIRT-1 distance (m) × 0.0084 + 36.4.

#### Exercise intensity

To ensure an objective evaluation of exercise intensity, HR was continuously monitored at 5-second intervals using Polar V800 devices with chest strap (Polar Electro Oy, Kempele, Finland) throughout each session. HRmean values for each repetition period were subsequently analyzed during data processing.

#### Cognitive function performance

Cognitive performance was assessed using the Stroop Color and Word Test – TBAG Form, which has been standardized and validated for the Turkish adult population. This test assesses core components of executive functioning, including selective attention, cognitive flexibility, processing speed, and particularly inhibitory control, which is challenged in the presence of conflicting stimuli^42^. Stimuli were presented as five standardized conditions displayed on a computer screen, and participants responded verbally according to the instructions. During administration, the examiner simultaneously followed the same TBAG scoring sheet and recorded responses in real time, marking errors and corrected responses in accordance with the test manual. The five conditions were: (1) reading color words in black ink, (2) reading color words in colored ink, (3) naming the colors of color patches (circles), (4) naming the ink color of neutral non-verbal stimuli (e.g., “XXXX”), and (5) naming the ink color of incongruent color words (e.g., the word “green” printed in red ink). Participants were asked to prioritize both speed and accuracy in their responses and to correct any errors as soon as they became aware of them. Completion time (s) from the start command to the final item was recorded for each condition; correction time was included in total time. In addition, error count and number of self-corrections were documented. Consistent with prior work, analyses focused primarily on completion time in the interference condition (Condition 5), which is closely related to common interference indices (e.g., B5–B3 or B5/B3). Completion time in Condition 5 was prioritized as the primary outcome, as time-based scores in the Stroop Test TBAG Form have been shown to be valid, reliable, and highly correlated with derived interference measures^42^. The test was administered in a quiet, well-lit room with participants seated at a desk, and a brief familiarization trial was performed before each session to minimize learning effects^43^.

#### Biochemical analysis

Venous blood samples (8 mL) were obtained from the antecubital vein with participants seated immediately before the session and immediately after the session. To minimize diurnal variation, all blood draws were performed within a fixed late-afternoon window (16:00–18:00). Samples were allowed to clot at room temperature for 30 min and were then centrifuged at 3000 × g for 15 min. Serum was aliquoted and stored at −80 °C until analysis.

Serum BDNF was quantified using a commercially available sandwich ELISA kit (SunRed Bio Company, Cat. No: 201-12-1303, intra-assay CV < 10%, Shanghai, China) following the manufacturer’s instructions. Before analysis, sera and reagents were brought to room temperature. All procedures were carried out under controlled laboratory conditions (22–24 °C, 40–60% relative humidity). The assay’s analytical range is 0.1–10 ng/mL with a lower limit of detection of 0.05 ng/mL, and all measurements fell within the validated range. To reduce between-run variability, samples were processed under identical conditions and analyzed within a single assay run. In line with the kit protocol, serum samples were assayed undiluted. Because of sample-volume constraints, each specimen was analyzed once as permitted by the manufacturer’s validated procedure. Optical density was read at 450 nm, and concentrations were derived from a standard curve using a four-parameter logistic model.

Blood lactate concentrations were determined with a Super GL2 analyzer (Müller Gerätebau GmbH, Freital, Germany), which operates using an enzymatic amperometric electrochemical principle. Calibration was performed before each measurement session in line with the manufacturer’s instructions. Samples were anonymized using coded labels, and the code key was revealed only after all laboratory analyses had been completed.

### Statistical analysis

Data are presented as mean ± SD. Analyses were conducted in Jamovi (v2.3). BDNF, lactate, Stroop completion times, and Stroop correction (number) were analyzed using linear mixed-effects models (LMM) implemented via the GAMLj module, including fixed effects of time (pre, post), condition (4 levels), their interaction (time × condition), period (1–4), and sequence (according to the randomization order), with a random intercept for participant. An independent covariance structure was assumed, and denominator degrees of freedom were estimated using the Satterthwaite approximation. HRmean, %HRmax and RPE values were analyzed using one-way ANOVA. Effect size metrics were selected and evaluated according to the specific statistical test applied. For the one-way ANOVA, partial eta squared ($\:{\eta\:}_{p}^{2}$) was calculated to determine the magnitude of main and interaction effects, interpreted as small (0.01), medium (0.06), and large (0.14). For the linear mixed-effects models, effect sizes for the post-hoc estimated marginal mean contrasts were reported as r values. The magnitude of r values was classified according to conventional thresholds as small (0.10), medium (0.30), and large (0.50)^44^. Statistical significance was set at *p* < 0.05. Data visualization was performed via JAMOVI (Version 2.3) and JASP (Version 0.19.3).

## Results

**Fig. 5:**
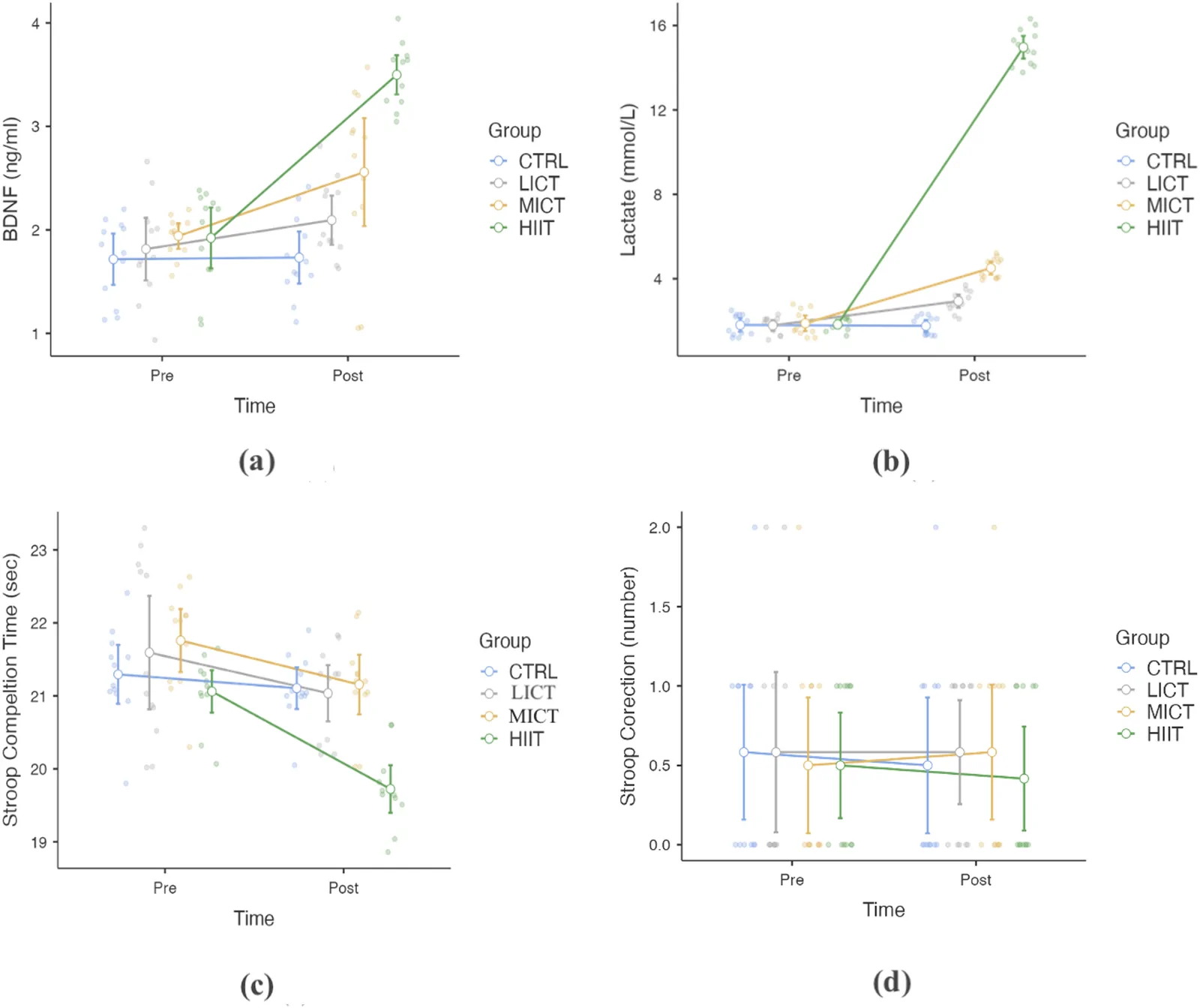
Individual and group-level data for serum BDNF, lactate, Stroop-5 completion time, and Stroop-5 correction numbers across the four experimental conditions (*n* = 12). (**a**) Serum BDNF levels (ng/mL) measured pre- and post-exercise; (**b**) Blood lactate levels (mmol/L) measured pre- and post-exercise; (**c**) Stroop-5 completion time (sec) measured pre- and post-exercise; (**d**) Stroop-5 correction numbers measured pre- and post-exercise. Individual data points are connected by lines to illustrate within-subject responses. *CTRL* control, *LICT* low-intensity continuous training, *MICT* moderate-intensity continuous training, *HIIT* high-intensity interval training, *BDNF* brain-derived neurotrophic factor, *ng/mL* nanograms per milliliter, *mmol/L* millimoles per liter, *sec* seconds.

In the following section of the present study, the statistical findings for BDNF, lactate, Stroop Test completion time, and Stroop Test correction score (number of corrections) across groups presented in Table 2; Fig. 5.

**Table 2:** BDNF, lactate and Stroop Test (completion time and correction scores) responses to experimental conditions (*n* = 12).

	CTRL		LICT		MICT		HIIT	
	Pre	Post	Pre	Post	Pre	Post	Pre	Post
	Mean ± SD	Mean ± SD	Mean ± SD	Mean ± SD	Mean ± SD	Mean ± SD	Mean ± SD	Mean ± SD
BDNF (ng/mL)	1.72 ± 0.39	1.73 ± 0.40	1.81 ± 0.48	2.10 ± 0.37	1.94 ± 0.19	2.56 ± 0.82	1.92 ± 0.46	3.50 ± 0.30
Lactate (mmol/L)	1.81 ± 0.50	1.77 ± 0.43	1.78 ± 0.39	2.94 ± 0.49	1.89 ± 0.58	4.51 ± 0.48	1.83 ± 0.85	14.97 ± 0.84
Stroop-5 completion time (sec)	21.30 ± 0.64	21.11 ± 0.45	21.59 ± 1.22	21.04 ± 0.61	21.76 ± 0.68	21.16 ± 0.64	21.06 ± 0.46	19.72 ± 0.51
Stroop-5 corrections (number)	0.58 ± 0.67	0.50 ± 0.67	0.58 ± 0.79	0.58 ± 0.51	0.50 ± 0.67	0.58 ± 0.67	0.50 ± 0.52	0.42 ± 0.51

*CTRL* control, *LICT* low-intensity continuous training, *MICT* moderate-intensity continuous training, *HIIT* high-intensity interval training, *BDNF* brain derived neurotrophic factor, *ng/mL* nanograms per milliliter, *mmol/L* millimoles per liter; *sec* second.

Figure 5a shows that the LMM indicated a significant main effect of time (before-after) on BDNF levels (F = 48.47, df = 1, df (res) = 69.81, *p* < 0.001). A significant main effect of condition was also observed (F = 7.92, df = 3, df (res) = 70.51, *p* < 0.001). In addition, the condition× time (before-after) interaction was significant (F = 14.57, df = 3, df (res) = 69.81, *p* < 0.001), demonstrating that the magnitude of pre–post BDNF change varied by exercise condition. Neither sequence (F = 1.05, df = 3, df (res) = 9.18, *p* = 0.414) nor period effects (F = 1.17, df = 3, df (res) = 72.35, *p* = 0.328) reached statistical significance. The participant-level random intercept was significant (*p* < 0.001). Pre-exercise BDNF levels did not differ between conditions (*p* > 0.05). Post-exercise comparisons, however, showed that HIIT elicited larger BDNF increases than CTRL (*p* < 0.001, *r* = 0.93), LICT (*p* < 0.001, *r* = 0.90), and MICT (*p* = 0.009, *r* = 0.61). Relative to CTRL, MICT also produced a greater increase (*p* = 0.002, *r* = 0.54). Within-condition (pre–post) contrasts derived from the LMM (Bonferroni-adjusted), expressed as before–after estimated marginal mean differences, indicated that no significant change was observed in the Control condition (mean difference = − 0.015, SE = 0.179, t(69.8) = − 0.086, *p* = 1.000) or in the LICT condition (mean difference = − 0.279, SE = 0.179, t(69.8) = − 1.563, *p* = 1.000). In contrast, significant pre–post changes were detected in the MICT condition (mean difference = − 0.618, SE = 0.179, t(69.8) = − 3.456, *p* = 0.026) and were more pronounced in the HIIT condition (mean difference = − 1.576, SE = 0.179, t(69.8) = − 8.819, *p* < 0.001) (Table 3).

Figure 5b summarises the LMM results, showing a significant main effect of time on lactate concentrations (F = 1538.00, df = 1, df(res) = 82, *p* < 0.001). A significant main effect of condition was likewise observed (F = 400.00, df = 3, df(res) = 82, *p* < 0.001). The time × condition interaction was also significant (F = 790.67, df = 3, df(res) = 82, *p* < 0.001), indicating that the magnitude of the pre–post lactate response differed across exercise conditions. Neither sequence (F = 0.471, df = 3, df(res) = 82, *p* = 0.703) nor period effects (F = 1.03, df = 3, df(res) = 82, *p* = 0.365) reached statistical significance. The participant-level random intercept was significant (*p* < 0.001). Baseline (pre-exercise) lactate values did not differ between conditions (*p* > 0.05). In contrast, post-exercise comparisons demonstrated that HIIT elicited greater lactate increases than CTRL (*p* < 0.001, *r* = 0.99), LICT (*p* < 0.001, *r* = 0.99), and MICT (*p* = 0.009, *r* = 0.99). Moreover, MICT produced larger increases than CTRL (*p* < 0.001, *r* = 0.95) and LICT (*p* < 0.001, *r* = 0.85). Relative to CTRL, LICT also resulted in a greater increase (*p* < 0.001, *r* = 0.76).

Figure 5c presents the LMM findings, demonstrating a significant main effect of time on Stroop completion time (F = 24.05, df = 1, df(res) = 82, *p* < 0.001). A significant main effect of condition was also evident (F = 4.64, df = 3, df(res) = 82, *p* = 0.005). The time × condition interaction also reached significance (F = 3.07, df = 3, df(res) = 82, *p* = 0.032), indicating that the extent of pre–post change varied by exercise condition. Sequence effects were not significant (F = 2.56, df = 3, df(res) = 82, *p* = 0.061), and no significant period effect was observed (F = 1.02, df = 3, df(res) = 82, *p* = 0.386). The participant-level random intercept was significant (*p* < 0.001). Baseline Stroop completion time did not differ between conditions (*p* > 0.05). Post-exercise comparisons, however, showed that only HIIT produced a significantly shorter (improved) Stroop completion time relative to CTRL (*p* = 0.008, *r* = 0.81), LICT (*p* = 0.003, *r* = 0.91), and MICT (*p* = 0.004, *r* = 0.89). Within-condition (pre–post) contrasts derived from the LMM (Bonferroni-adjusted), expressed as before–after estimated marginal mean differences, indicated that no significant change was observed in the Control condition (mean difference = − 0.148, SE = 0.301, t(57.2) = − 0.491, *p* = 1.000), the LICT condition (mean difference = − 0.573, SE = 0.285, t(57.2) = − 2.008, *p* = 1.000), or the MICT condition (mean difference = − 0.088, SE = 0.285, t(57.2) = − 0.308, *p* = 1.000). In contrast, a significant reduction in Stroop completion time was observed in the HIIT condition (mean difference = 2.159, SE = 0.285, t(57.2) = 7.567, *p* < 0.001), indicating an improvement in executive function performance following HIIT.

Figure 5d shows that the LMM did not yield a significant main effect of time on Stroop correction (F = 0.029, df = 1, df (res) = 67.8, *p* = 0.866). The main effect of condition was also not statistically significant (F = 0.081, df = 3, df (res) = 69.8, *p* = 0.970). In addition, the time × condition interaction was not statistically significant (F = 0.1055, df = 3, df (res) = 67.8, *p* = 0.957), showing that the magnitude of pre–post changes did not differ across exercise conditions. Sequence effects were not statistically significant (F = 0.0856, df = 3, df (res) = 14.5, *p* = 0.967), and period effects likewise did not reach significance (F = 0.8235, df = 3, df (res) = 71.5, *p* = 0.485). According to the analysis, there was no significant difference in pre- and post-exercise Stroop correction between the groups (*p* > 0.05).

**Fig. 6:**
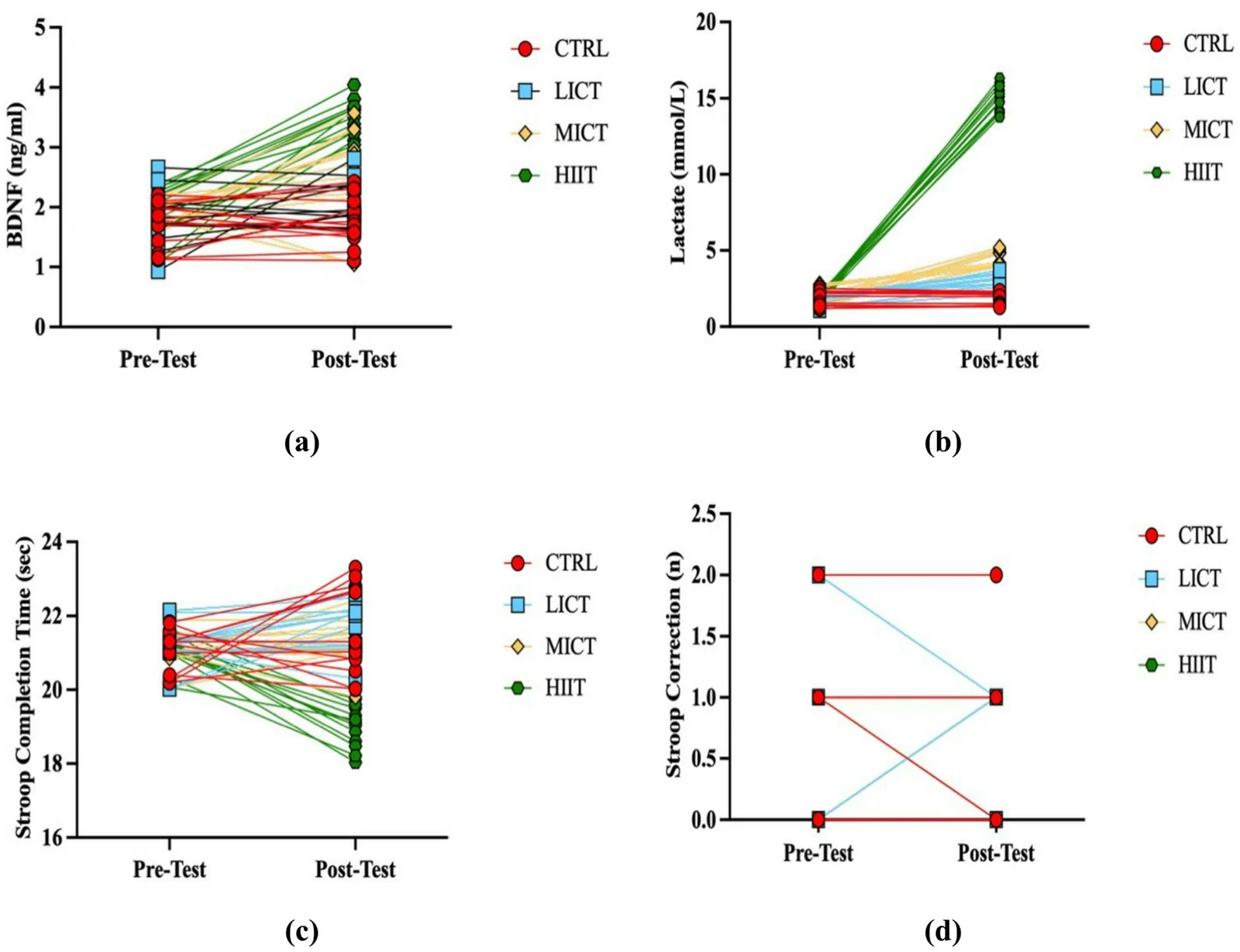
Inter-individual pre-to-post trajectories of serum BDNF, blood lactate, Stroop completion time, and correction score across conditions (*n* = 12). *CTRL* control; *LICT* low-intensity continuous training, *MICT* moderate-intensity continuous training, *HIIT* high-intensity interval training, *BDNF* brain-derived neurotrophic factor, *ng/mL* nanograms per milliliter, *mmol/L* millimoles per liter, *sec* seconds.

Figure 6 illustrates individual pre-to-post changes in serum BDNF, blood lactate, Stroop completion time, and correction score across the experimental conditions.

**Fig. 7:**
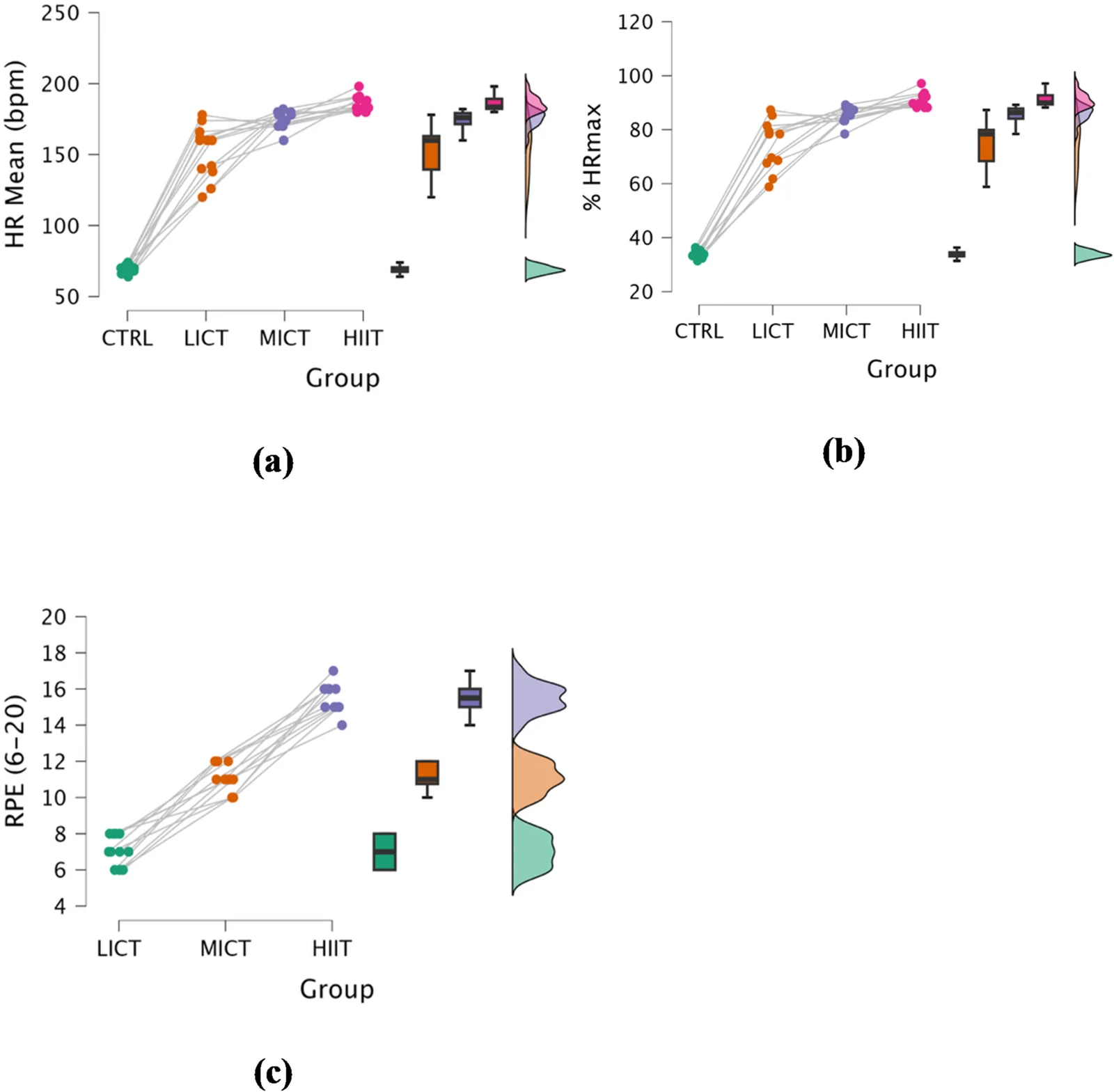
Figure presents the heart rate mean (HRmean) values and rating of perceived exertion (RPE) scores for the CTRL, LICT, MICT, and HIIT groups (*n* = 12). (**a**) HR mean values measured during the exercise are shown; (**b**) %HRmax values measured during the exercise are shown (**c**) RPE scores measured post-exercise are shown. *BDNF* brain derived neurotrophic factor, *bpm* beat per minute, *CTRL* control, *HIIT* high-intensity interval training, *LICT* low-intensity continuous training, *MICT* moderate-intensity continuous training.

In the following section of the present study, the statistical findings for HRmean, %HRmax, and RPE across groups are presented in Table 3 and Fig. 7.

**Table 3:** Heart rate and rating of perceived exertion responses to experimental conditions (*n* = 12).

	CTRL	LICT	MICT	HIIT
	Mean ± SD	Mean ± SD	Mean ± SD	Mean ± SD
HR mean (bpm)	68.92 ± 2.90	152.17 ± 18.60	174.75 ± 6.70	185.80 ± 5.50
%HRmax	33.83 ± 1.40	74.58 ± 9.11	85.50 ± 3.030	91.04 ± 2.72
RPE (A.U.)	-	7 ± 0.90	11.08 ± 0.80	15.50 ± 0.80

*A.U.* arbitrary unit, *bpm* beat per minute, *HIIT* high-intensity interval training, *HRmean* heart rate mean, *LICT* low-intensity continuous training, *MICT* moderate-intensity continuous training, *RPE* rating of perceived exertion.

HRmean and RPE increased progressively across LICT, MICT, and HIIT, confirming the successful manipulation of exercise intensity. Consistent with this pattern, the results of the analyses for HR mean (Fig. 7a) indicated a statistically significant difference between the groups (df = 3, F = 3177.20, *p* < 0.001, η²_p_= 0.966). Post-hoc analyses further revealed that LICT showed significantly greater values than the CTRL (*p* < 0.001; CI95%: 67.22, 99.28). Moreover, the MICT and HIIT demonstrated significantly higher values than the LICT (*p* = 0.013, CI95%: 4.29, 40.88; *p* < 0.001, CI95%: 15.17, 52 respectively). Both MICT and HIIT were higher than CTRL (*p* < 0.001, CI95%: 98.66, 113.01; *p* < 0.001, CI95%: 110.73, 122.94 respectively), and the HIIT showed significantly higher values than the MICT (*p* = 0.008, CI95%: 2.71, 19.30).

The %HRmax values differed significantly across conditions (Fig. 7b). One-way ANOVA revealed a significant main effect of condition, F(3, 33) = 316.11, *p* < 0.001, η²*p* = 0.966. Mean %HRmax increased progressively across conditions, with values of 33.83 ± 1.40% in CTRL, 74.58 ± 9.11% in LICT, 85.50 ± 3.03% in MICT, and 91.04 ± 2.72% in HIIT.

Bonferroni-adjusted post hoc comparisons showed that all conditions differed significantly from each other. %HRmax was significantly higher in LICT than in CTRL, mean difference = 40.80, SE = 2.45, t(11) = 16.65, *p* < .001; higher in MICT than in CTRL, mean difference = 51.87, SE = 1.10, t(11) = 47.31, *p* < .001; and higher in HIIT than in CTRL, mean difference = 57.26, SE = 0.93, t(11) = 61.46, *p* < .001. MICT was also significantly higher than LICT, mean difference = 11.07, SE = 2.80, t(11) = 3.95, *p* = .014. HIIT was significantly higher than LICT, mean difference = 16.46, SE = 2.81, t(11) = 5.85, *p* < .001, and MICT, mean difference = 5.39, SE = 1.27, t(11) = 4.25, *p* = .008. These findings verify that the protocols generated distinct low-, moderate-, and high-intensity cardiovascular demands.”

The results of the analyses for RPE, (Fig. 7c) indicated a statistically significant difference between the groups (df = 2, F = 216.87, *p* < 0.001, η²_p_= 0.961). Post-hoc analyses further revealed that the MICT demonstrated significantly higher values than the LICT (*p* < 0.001; CI95%: 3.14, 5.03), and the HIIT showed significantly higher values than both the LICT (*p* < 0.001, CI95%: 7.32, 9.68) and MICT (*p* < 0.001, CI95%: 3.47, 5.37).

## Discussion

This study investigated the acute effects of HIIT, MICT, and LICT on serum BDNF, lactate levels, and cognitive performance in healthy young males. As hypothesized, HIIT exerted significantly greater increases in both BDNF and lactate compared with other conditions. Additionally, a single HIIT session led to superior post-exercise performance on the Stroop interference task. While MICT produced moderate elevations in BDNF and lactate, neither LICT nor the resting condition yielded significant changes. These findings offer valuable insights into the potential coupling between BDNF and lactate stimulated by higher-intensity exercise. Our findings are consistent with previous human studies suggesting that HIIT facilitates neurotrophic and metabolic adaptations that underpin improvements in executive function^32,45,46^. Nonetheless, the optimal exercise modality and intensity for maximizing acute cognitive benefits remains unresolved, and elucidating the underlying neurobiological pathways is a major challenge^29,47^.

In line with our findings, numerous reviews^24,26,27^ and randomized controlled studies^48–51^ have reported that HIIT robustly elevates BDNF levels. Moreover, a converging body of evidence indicates that acute elevations in circulating BDNF are generally larger following higher relative intensities, with HIIT often producing more pronounced responses than continuous moderate- or low-intensity exercise. Across controlled studies, the direction of effect is broadly consistent with an intensity–dose relationship, although the magnitude of change varies with protocol design (e.g., interval structure), participant fitness, and sampling time points. Our results align with this pattern, showing the largest acute increase in serum BDNF following HIIT, while the lower-intensity conditions produced smaller or negligible changes^52–57^. Notably, the literature is not fully consistent, and discrepancies may partly reflect how high intensity is operationalized. For example, although Fernández-Rodríguez et al.^25^ reported that HIIT may be more effective than moderate-intensity exercise in enhancing BDNF levels, their classification of ‘high-intensity’ (~ 75% VO₂max) may not align with more stringent definitions of HIIT (> 85% VO₂max), thereby limiting the interpretability of the findings. Similarly, our previous research in sub-professional athletes found no acute BDNF elevation following either game-based or running-based HIIT interventions^58^, suggesting that population characteristics, training status, or protocol-specific variables may influence the neurotrophic response. Accordingly, not all studies support a superior HIIT effect on acute BDNF elevations^29^.

HIIT is known to engage anaerobic energy systems, resulting in transient cerebral hypoxia and increased production of metabolites and signaling agents such as reactive oxygen species, lactate, irisin, and cathepsin^3,45,59–61^. Experimental work suggests that exercise-induced shifts in cellular energy balance and redox state may activate transcriptional programs that are compatible with increased BDNF expression^24,62,63^. Lactate, a molecule once considered merely a metabolic waste product, has emerged as a potential signaling molecule that may be involved in the regulation of BDNF expression^64^. Intense exercise may also be associated with a metabolic substrate shift in the brain (glucose to lactate) as the preferred energy source. The brain may utilize lactate to help meet the elevated energy demands needed to sustain neuronal function. This adaptation may support neuronal energy demands under hypoxic stress and has been proposed to reflect lactate’s dual role as both a metabolic fuel^16,65^ and a neuromodulatory signal. Collectively, these mechanisms are consistent with a potential role of lactate-related signaling in BDNF-associated neurobiological responses during and after HIIT^3,13,66,67^. While these mechanisms provide biological plausibility, the present study was not designed to isolate pathway-specific contributions, and therefore causal inferences should be avoided.

Based on the proposed mechanisms, we can speculate that the parallel increases in lactate and BDNF observed in our study following HIIT support the hypothesis that lactate may act as a mediator of neurotrophic adaptations to intense exercise. This may also reflect the study design, as sessions were matched for duration but not for physiological load, which was an intentional feature and may have partly contributed to the greater lactate and BDNF responses observed following HIIT. In line with our findings, Ferris et al.^52^ reported that BDNF responses are more strongly associated with exercise intensity rather than duration and are moderately correlated with post-exercise lactate concentrations. Similarly, Gibbons et al.^53^ demonstrated that the markedly greater BDNF response observed following a HIIT protocol, compared to prolonged low-intensity exercise, was significantly correlated with elevated circulating lactate levels. Despite being conducted in a clinical population, Boyne et al.^48^ showed that a 20-minute HIIT session led to simultaneous increases in circulating BDNF and lactate, supporting the link between metabolic intensity and neuroplastic adaptations. However, contrasting evidence from other studies suggests that elevated lactate levels alone may not consistently predict BDNF responses across different exercise modalities or participant populations^68^. This may, in fact, help explain why, despite lactate levels being significantly higher after MICT than after LICT and CTRL, the concomitant post-MICT increase in BDNF did not reach statistical significance relative to those conditions. This discrepancy underscores the complexity of the lactate–BDNF relationship and indicates the need for further investigation to clarify its mechanistic underpinnings.

HIIT is a time-efficient training method involving short bursts of intense activity interspersed with recovery periods, yet its cognitive and psychological benefits are not fully understood^61,69^. Supporting one of our main hypotheses, improved task performance in the incongruent condition, consistent with enhanced inhibitory control, was observed only following the HIIT condition, as indicated by shorter response times on the Stroop Test. Importantly, this faster performance was not accompanied by an increase in correction errors, suggesting that the improvement in performance was not achieved at the expense of accuracy. However, the functional relevance of the approximately 6% reduction in completion time should be interpreted cautiously, as no established minimal clinically important difference has been defined for Stroop performance in healthy young adults. These results suggest that even an acute exercise session can temporarily improve executive function performance^70,71^. Recent research has concluded that both HIIT and MICT can improve inhibitory control in healthy adults, with no significant difference observed among these modalities^2^, other studies have reported that improvements in executive function are more pronounced following HIIT compared to MICT^18,30,71^. Ferris et al.^52^ were the first to conclude that HIIT had a greater influence on cognitive function (Stroop color-word score) than MICT, and this finding has since been replicated by numerous studies. Kujach et al.^21^ found improved Stroop test performance following sprint interval exercise, while Ballester et al.^29^ reported that inhibitory control benefits emerged only after HIIT and were most evident when the task was administered 15 min post-exercise, emphasizing the critical role of intensity and timing. Consistent with these observations, Buzdağlı et al.^57^ also demonstrated that while both HIIT and MICT improved cognitive performance, HIIT led to superior outcomes in Stroop task reaction time and accuracy under the incongruent condition—suggesting a greater impact on inhibitory control.

The cognitive enhancements observed after acute HIIT are likely driven by multiple interrelated mechanisms, involving neuroelectric, vascular, metabolic, and neurochemical pathways. Previous research has indicated that acute HIIT may enhance executive function by promoting greater neuroelectric efficiency, accelerating stimulus processing^72^, and improving oxygen delivery to the prefrontal cortex^73^. Moreover, HIIT may transiently increase oxidative and inflammatory responses that can influence prefrontal cortex activity, which is central to executive functioning^74^. In addition, BDNF is known to support cognitive function by enhancing synaptic transmission, facilitating long-term potentiation, and promoting neurotransmitter release^75^. Consistent with this, both animal and human studies have shown that peripheral BDNF levels are positively associated with cortical BDNF concentrations^59^ and correlate with improved cognitive performance following acute exercise^76^. Lactate represents another plausible contributor, acting as an oxidative substrate and signaling molecule; experimental and human studies have proposed that lactate dynamics may influence brain energetics and neurotrophic regulation following intense exercise^21,66,77^. In this context, the HIIT-specific improvement in Stroop performance in our study is compatible with a framework in which higher exercise intensity elicits greater lactate accumulation^68^, alongside larger BDNF responses, which together may support transient gains in executive function^78,79^. In addition, Chaney et al.^80^ reported that Stroop task improvements after electrical muscle stimulation were positively correlated with lactate increases, providing complementary non-exercise evidence for a possible lactate–cognition relationship. Notably, cognitive assessments in the present study were conducted immediately after exercise, whereas some studies suggest that peak cognitive benefits may occur following a brief post-exercise delay^81,82^. Accordingly, as central uptake and pathway-specific mediators were not assessed, these mechanistic interpretations remain indirect, and other exercise-induced factors (e.g., irisin, IGF-1, VEGF) may also contribute^83^.

### Limitations

The present findings should be interpreted in light of several limitations. First, the relatively small sample, comprising only healthy young men, may restrict the applicability of the findings to broader populations. In addition, because the a priori power calculation was based on a large effect size derived from a single previous study, the required sample size may have been optimistically estimated. Accordingly, the study may have had limited statistical power to detect smaller effects, particularly for cognitive outcomes such as Stroop error scores. Given known sex-related differences in hormonal status, BDNF regulation, and cognitive responses to exercise, these findings should not be generalized to women without further investigation. Second, although serum BDNF is widely used as a peripheral marker, it may not directly reflect central nervous system BDNF activity; therefore, conclusions regarding underlying neurobiological mechanisms should be interpreted with caution. Third, the trial was registered retrospectively rather than prospectively, which may limit transparency regarding the pre-specified study protocol. Additionally, executive function was assessed with a single cognitive task^69^, which may limit the ability to capture the multidimensional nature of executive functioning and reduce the generalizability of the findings across different cognitive domains. Lactate measurements were limited to peripheral blood, precluding direct inference about cerebral lactate dynamics or causal pathways. Moreover, within-participant associations between changes in lactate and BDNF were not examined, and should be addressed in future studies with larger samples. Finally, biomarker assessments were conducted only immediately after exercise, which may not fully capture the temporal dynamics of BDNF responses^84^.

### Future research directions

Future research should recruit more heterogeneous samples, including women, older individuals, and clinical populations, and account for sex-specific factors such as menstrual cycle phase and hormonal contraceptive use. Future studies should also employ multiple post-exercise time points to better characterize BDNF kinetics. Mechanistic designs that combine peripheral biomarkers with neuroimaging or neurophysiological measures may help disentangle central versus peripheral contributions. Finally, future studies could investigate dose–response relationships between exercise intensity, lactate production, and cognitive outcomes to refine evidence-based exercise prescriptions for brain health.

### Practical and translational implications

The present findings suggest that HIIT may represent a time-efficient approach to acutely enhance executive function performance in healthy young adults. Given its brief duration and associated neurochemical responses, HIIT may be relevant for settings in which time constraints limit prolonged exercise sessions. However, further research is needed to determine whether these acute responses translate into meaningful and sustained neuroplastic or functional benefits and to examine associations among BDNF, lactate, and Stroop performance in larger samples.

## Conclusions

A single HIIT session elicited greater acute increases in serum BDNF and improved executive-function performance compared with MICT and LICT in healthy young males. While MICT induced modest neurochemical changes relative to CTRL, only HIIT was accompanied by a measurable cognitive benefit. These results support HIIT as a potent acute stimulus for neurochemical and cognitive responses, a pattern consistent with a role for lactate–BDNF coupling.

## Data Availability

The datasets generated and/or analysed during the current study are not publicly available due to ethical and privacy considerations to protect participant identity but are available from the corresponding author on reasonable request.

## Abbreviations

BDNF: Brain-derived neurotrophic factor
HIIT: High-intensity interval training
IGF-1: Insulin-like growth factor
LICT: Low-intensity continuous exercise
MAS: Maximal aerobic speed
MICT: Moderate-intensity continuous exercise
RPE: Ratings of perceived exertion
VEGF: Vascular endothelial growth factor
VO_2_max: Maximal oxygen uptake
YYIRT-1: Yo-Yo Intermittent Recovery Test Level-1
